# Advancements in Facial Reconstruction Surgery: The Role of Bioengineered Materials, Artificial Intelligence, and 3D Printing in Addressing Economic Disparities

**DOI:** 10.7759/cureus.89627

**Published:** 2025-08-08

**Authors:** Khushi A Murnal, Daniel S Knupflemacher, Paola A Chavez, Vishwas Karanth, Aura C Luzardo, Rafael Giner, Erika Aguirre, Maitri Patel, Ramsha Ali

**Affiliations:** 1 Medicine, Belgaum Institute of Medical Sciences, Belgaum, IND; 2 Medicine, Westhill University, Mexico City, MEX; 3 Plastic Surgery, Universidad Peruana Cayetano Heredia, Lima, PER; 4 Medicine, Subbaiah Institute of Medical Sciences, Shimoga, IND; 5 Surgery, Universidad de Buenos Aires, Buenos Aires, ARG; 6 Surgery, Universidad Central de Venezuela, Caracas, VEN; 7 Medicine, Universidad Autónoma de Guadalajara, Zapopan, MEX; 8 Medicine, The College of New Jersey, Ewing Township, USA; 9 Medicine and Surgery, Peoples University of Medical and Health Sciences, Nawabshah, PAK

**Keywords:** 3d imaging, acellular dermal matrix, craniofacial surgery, decellularization-recellularization, facial plastic reconstructive surgeries, flap techniques, plastic surgery, radiofrequency, regenerative medicine, tissue engineering

## Abstract

The advancement of science and technology is an undeniable phenomenon that is progressively transforming all aspects of human life, including scientific, social, humanitarian, and environmental fields, among others. Facial reconstruction surgery has recently gained much attention owing to the incorporation of new technologies, such as bioprinting, regenerative medicine (RM), and artificial intelligence (AI) in surgery. These advancements have led to more innovative, site-specific, and optimal methods of addressing the challenges of facial reconstruction following trauma, congenital malformations, and oncological resections. However, this progress is not available in many underprivileged parts of the world, and it is significantly limited to certain populations with high economic resources. This review is designed to offer a thorough evaluation of the state of the art in facial reconstruction surgery, with regard to the deployment of bioengineered materials, AI-based surgical planning, and three-dimensional (3D) printing. It also analyzes how the most advanced techniques in facial reconstructive surgery are being implemented for the benefit of the population and how this implementation is affected by the economic disparities of each society. Facial reconstruction surgery is rapidly changing, and technology is playing a key role in this change. Nevertheless, innovation alone is not enough. For this, the effectiveness of innovation has to be combined with the best practices of implementation, which requires a multidisciplinary effort between clinicians, researchers, and policymakers, to ensure the safety, accessibility, and ethical integrity of advancements. The success of facial reconstruction surgery cannot be attributed to technological sophistication alone, but also to the frequency of improvement in the quality of life.

## Introduction and background

Many people around the world seek to reflect their self-esteem through physical beauty, with the face being one of the most significant aspects [1]. The first impression we convey as humans is often determined by our face and facial expressions [2]. Those who have a face that aligns with their society’s beauty standards are frequently considered fortunate, but what happens when that is not the case? It is a complex question that emphasizes self-esteem, beauty objectives, and attachment to society standards.

Facial reconstruction is a specialized and extensive field that plays a crucial role in restoring both functionality and aesthetics for those born with congenital deformities, suffering from pathological conditions, or who have experienced trauma affecting their face [3]. To bridge the gap and provide access to reconstructive surgeons in resource-deficient areas, a significant part of international work in facial plastic reconstructive surgery involves humanitarian missions instead of structured research and education [4]. Although there has been significant improvement in regenerative medicine (RM), almost all technical approaches still fall short in achieving the needed construction of proper biological, structurally steady, and functional regenerated tissues and organs. The decellularization-recellularization (D/R) technique creates functional and bioengineered tissues from existing donor or artificial scaffolds, which can be an excellent strategy to overcome this obstacle [5].

The postoperative results of early debridement and advancements in plastic surgery techniques have not been fully assessed qualitatively using the Scar Cosmesis Assessment and Rating (SCAR) scale, although they have demonstrated effective treatment in facial scars caused by trauma [6]. An increasing number of research studies point to stem cells as an optimistic innovation in disease modeling, regenerative medicine, and screening of drugs [7].

There is a considerable amount of contribution to craniofacial reconstruction and facial aesthetics for the improvement of scientific literature, guiding future studies/research, and molding innovative clinical applications in the field of plastic surgery, biomedical engineering, and regenerative medicine [8]. Challenges in craniofacial reconstruction surgeries due to the intricacy of facial anatomy, functional demands, and aesthetic importance play an important role in providing the needed outcome [8]. For instance, forehead reduction surgery is commonly sought by individuals with a high or elongated forehead, often due to genetics, aging, or hairline recession, with the face being the area that requires optimal results [9]. Standardizing medical photography in facial plastic surgery by developing a compact, cost-effective, and efficient imaging system plays a critical role in patient assessment, treatment planning, surgical documentation, and outcome evaluation [10], especially when comparing treatment effectiveness. Therefore, photography should continue to play an important role in primary evaluation and follow-up. Subsurface radiofrequency (RF) treatments enhance the credibility of non-invasive RF treatments in plastic surgery and provide evidence and guidance on using RF technology more effectively and safely; however, their clinical effectiveness remains unproven when compared to traditional surgical methods [11].

Addressing barriers that might limit global implementation of emerging technologies in regenerative medicine, biocompatible biomaterials, and artificial intelligence (AI) that enhance the efficacy, accessibility, and innovation of modern facial reconstruction techniques, the "humanitarian trips" have gained popularity as an approach to address the shortage of reconstructive surgeons in resource-limited areas. The international work in facial plastic and reconstructive surgery (FPRS) includes humanitarian work that highlights inequality and advocates for more inclusive research practices that involve professionals from low- or middle-income countries (LMICs), attempting to build on the quality of FPRS research worldwide [4]. Emerging technologies, such as the application of the decellularization-recellularization technique in plastic and reconstructive surgery, could potentially transform tissue engineering (TE) and regenerative medicine by giving an alternative solution to traditional grafting and transplantation techniques [2]. Evidence-based growth in facial trauma treatment and scar management in plastic and reconstructive surgery offers quantitative data and standardized assessment methods that could be valuable for future research and clinical practice in these fields [6].

The advancements that bioprinting and stem cell therapy have reached show a glimpse of the future of craniofacial reconstructions: more customized to individual patient needs; less invasive, reducing recovery time and complications; and more accessible and affordable over time [7].

Multiplane forehead shortening significantly contributes to facial aesthetics, enhances patient safety, and drives surgical innovation [8]. There are newer surgical techniques in forehead reduction that show a refined multiplane approach that fit the aesthetic objectives, along with the principles of preserving nerve function and muscle integrity [9]. Improvements in patient care through medical photography can be achieved through accurate and consistent medical documentation, which can be a valuable tool in medical education and teaching resources [10].

The inventive technique of decellularization-recellularization provides customized tissue regeneration while minimizing the risk of immune rejection by allowing a patient's own cells to repopulate scaffolds [5]. By demonstrating both aesthetic and symptomatic benefits, it helps enhance both patient satisfaction and quality of life post-facial trauma [6]. Human-induced pluripotent stem cells (HiPSCs) play a pivotal role in underscoring both opportunities and challenges; they even offer valuable perception for researchers and clinicians looking forward to incorporating regenerative medicine approaches into clinical practice [7]. By advancing the unification of tissue engineering (TE) and regenerative medicine (RM), these findings have a chance to transform craniofacial reconstruction and improve aesthetic outcomes, along with influencing the evolution of future surgical techniques [8]. Vast databases contribute to the growing field of minimally invasive and patient-specific plastic surgery methods while also linking cosmetic improvements and functional preservation [9]. Medical photography in facial plastic surgery benefits surgeons, researchers, patients, and educators by providing cost-effective, high-quality, and consistent imaging [10]. Facial reconstruction surgery has made remarkable progress in recent years, thanks to the evolution of microsurgery, new materials, and regenerative medicine. These innovations have also enhanced the functional and cosmetic results for people with trauma, congenital anomalies, and tumor resections [3]. This review aims to offer a systematic assessment of the state of the art in reconstructive methods, against the background of their efficacy, as well as existing difficulties and deficiencies.

The use of biocompatible materials is one of the most significant developments in the field. Autologous tissue grafts are still the preferred method, but the literature suggests that acellular dermal matrix (ADM) and bioengineered scaffolds can provide comparable results with better graft incorporation and a lower rate of rejection [12]. Furthermore, three-dimensional (3D) printed implants are gradually becoming more sophisticated and can create patient-matched constructs that not only improve the fit but also the strength [13].

This review will also focus on the implications of AI in surgical planning and patient management. Some studies have proposed the application of AI models to analyze images, forecast the possible outcome of surgery, and help in the assessment of patients after the operation [14]. In addition, the integration of machine learning (ML) techniques with electronic health records (EHRs) is starting to define more personalized care pathways, which include the optimization of both short-term and long-term plans for procedures and treatments, respectively.

However, there are several issues that prevent the full implementation of AI and new biomaterials in reconstructive surgery. Problems with data, moral concerns, and legal issues remain a challenge that delays the adaptation of approaches to clinical practice [15]. Moreover, another problem is ensuring the generalizability of AI-based models and their reliability across multiple patients.

**Figure 1:**
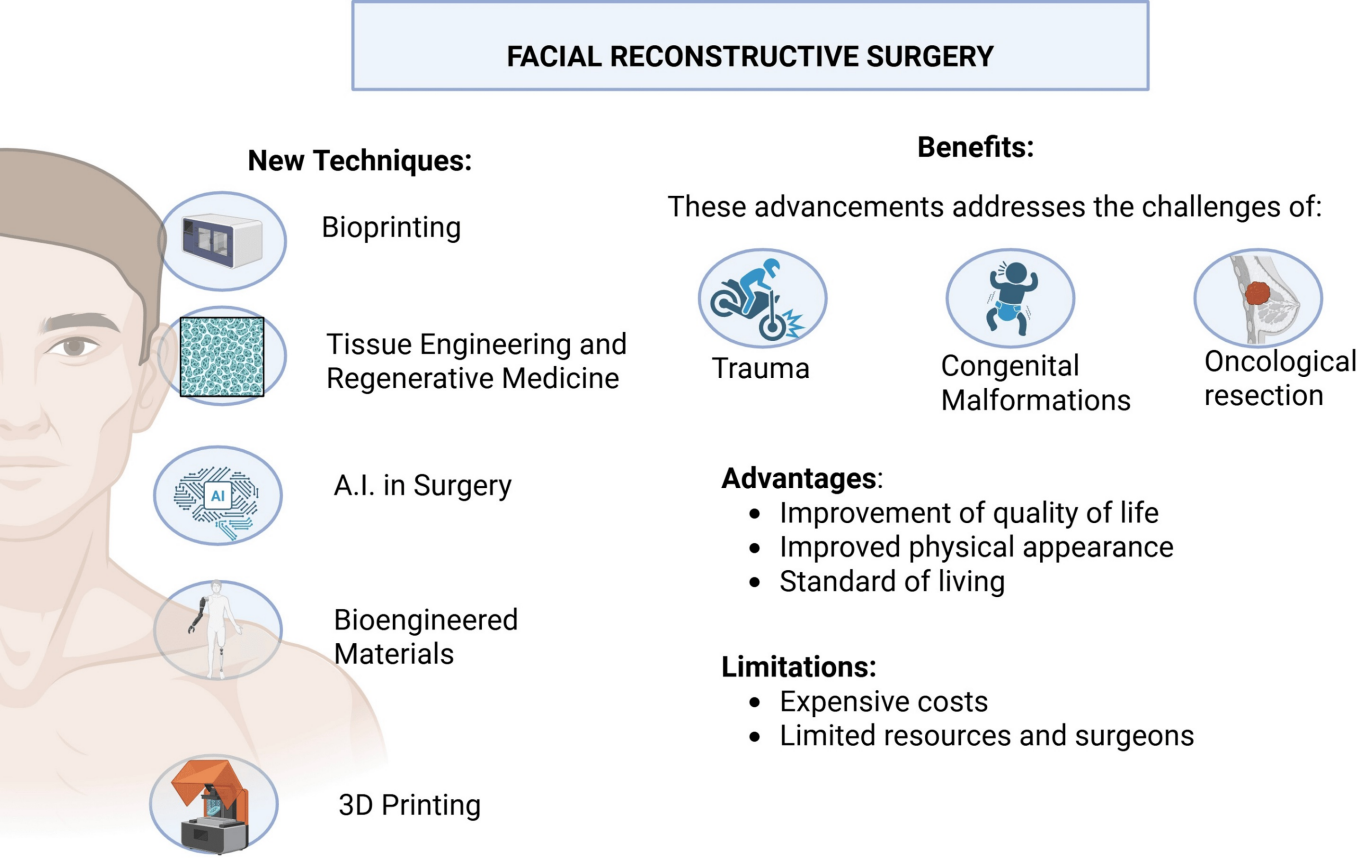
New techniques in facial reconstructive surgery and their benefits The upcoming newer techniques in facial reconstructive surgery and their benefits, advantages, and limitations. These techniques included bioprinting, tissue engineering and regenerative medicine, AI in surgery, bioengineered materials, and 3D printing. AI: artificial intelligence Created in BioRender by Zain Abdin (2025) (https://BioRender.com/nvm8u28)

This review explores the current information in facial reconstruction techniques, the latest emerging technologies, and the impact of regenerative medicine on outcomes (Figure 1), and how these advancements might influence the future of surgical techniques and reconstructive options. A thorough analysis might also establish a new field for further investigation.

This narrative review primarily includes studies published within the last 10 years, with a few exceptions (3-4 studies) that were deemed highly relevant to the topic. The inclusion criteria were as follows: human studies involving individuals aged 18 years and older, published in English, and aligned with the PICO (Patient or Problem, Intervention, Comparison, and Outcome) framework. Exclusion criteria included studies involving pregnant individuals, non-human subjects, and pediatric populations, and articles published beyond the 10-year window that did not meet the relevance threshold.

## Review

**Figure 2:**
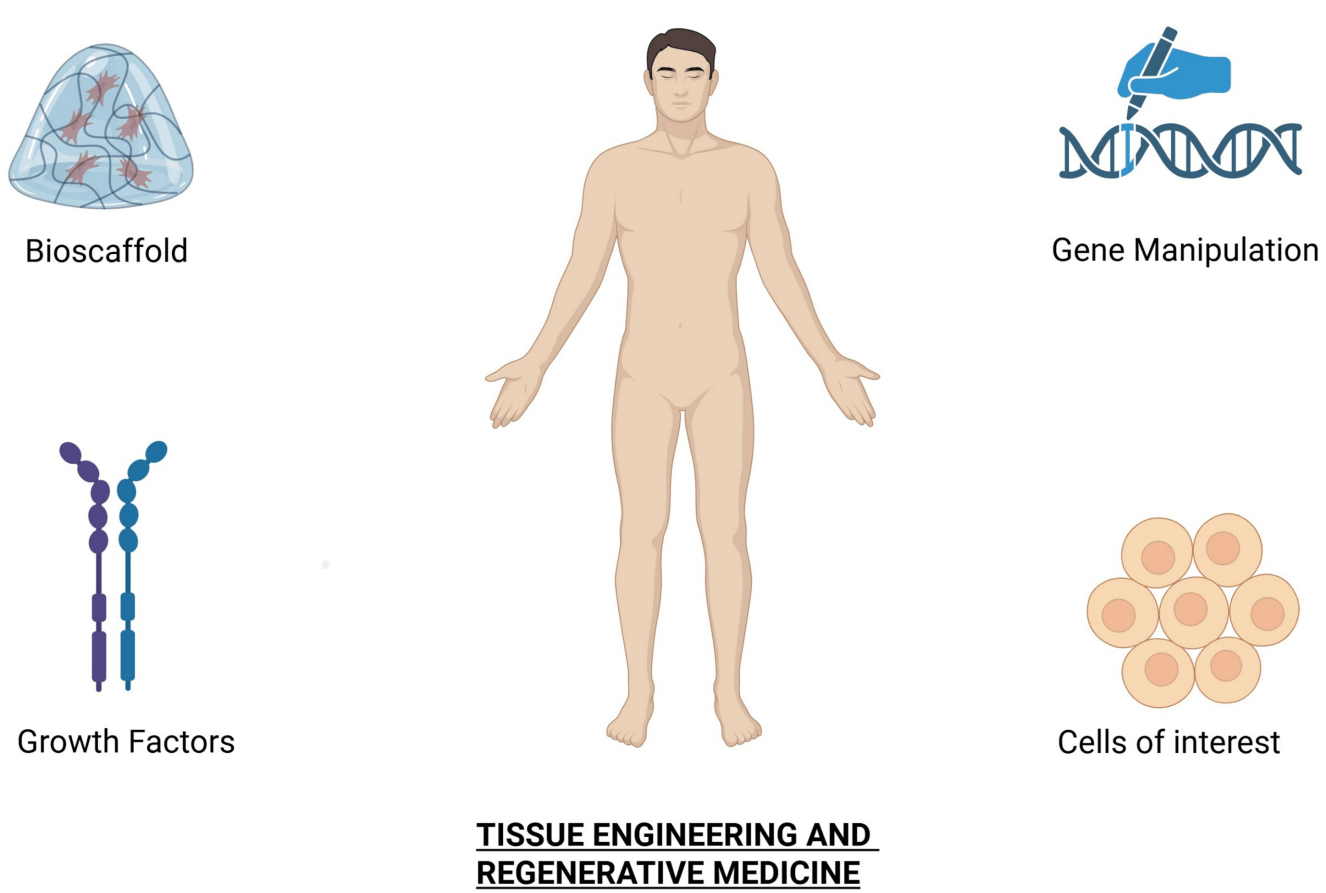
Tissue engineering and regenerative medicine alternatives The figure displays the components of tissue engineering: bioscaffolding, gene manipulation, growth factors, and cells of interest. Created in BioRender by Zain Abdin (2025) (https://BioRender.com/ubku6i7)

Tissue engineering (TE) and regenerative medicine (RM) have emerged as promising alternatives to traditional grafting techniques in craniofacial reconstruction and facial aesthetics. These approaches facilitate natural tissue regeneration by incorporating cells, natural or synthetic scaffolds, growth factors (GFs), gene manipulation, or combinations of these components (Figure 2). They aim to restore various tissue types, including bone, cartilage, soft tissue, nerves, and blood vessels, damaged by congenital anomalies or acquired defects while minimizing the need for immunosuppression [8].

Despite significant advances and encouraging results from both preclinical and clinical studies, the successful vascularization of TE constructs remains a primary limiting factor. Adequate tissue perfusion is essential for development and host integration, especially when dealing with large volumes or whole organs [8].

A notable innovation in this field is the use of acellular dermal matrix (ADM). ADM is a dermal graft in which the epidermis and all cellular elements are removed to reduce immunogenicity. It has a wide range of applications in plastic and reconstructive surgery, including the reduction of donor site morbidity, improved wound healing, and enhanced outcomes in soft tissue reconstruction [16].

**Figure 3:**
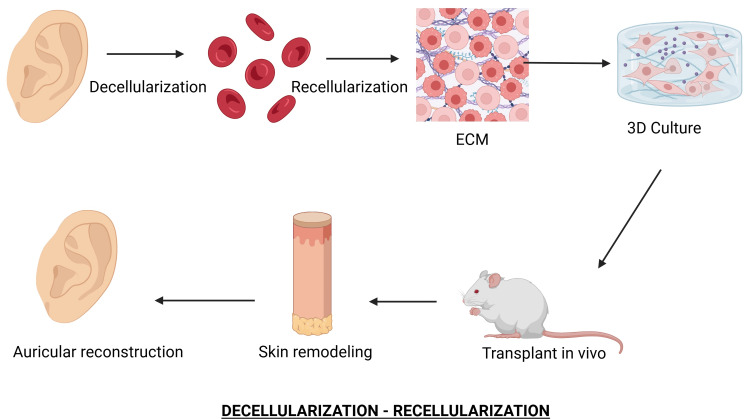
Decellularization-recellularization This process involves the ECM and the other components to reconstruct the auricular tissue. ECM: extracellular matrix Created in BioRender by Zain Abdin (2025) (https://BioRender.com/iw0q38f)

Another advanced method is the decellularization-recellularization (D/R) technique. This involves removing antigenic components from donor tissue and repopulating the resulting extracellular matrix (ECM) scaffold with autologous cells (Figure 3). Recent developments have optimized decellularization protocols and scaffold design, preserved vascular networks, and facilitated tissue regeneration with enhanced functionality [5].

Complementary to these advances, resident stem cells have been identified in structures such as the lacrimal gland, corneal limbus, orbital fat, and extraocular muscles, offering potential for in vitro disease modeling and regenerative transplantation [17]. Human-induced pluripotent stem cells (HiPSCs) have shown the ability to differentiate into a variety of tissues, including the skin, vasculature, and nerves. In some cases, HiPSC-derived neurons have restored sensation following facial nerve injury [7].

Three-dimensional printing is another transformative tool in craniomaxillofacial surgery. A systematic review categorized its direct surgical applications into four types: contour models, surgical guides, splints, and implants. Patient-specific 3D printing enhances the precision of craniofacial reconstruction, reduces operative time, and improves both aesthetic and functional outcomes [18].

While reconstructive procedures are critical, aesthetic results and patient satisfaction are also strongly influenced by non-surgical interventions. Minimally invasive treatments such as Botox injections, dermal fillers, and permanent makeup have substantial effects on patients' perceptions and emotional well-being [12].

**Figure 4:**
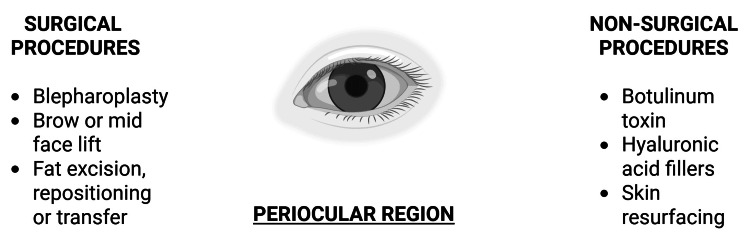
Rejuvenation of the periocular region Surgical and non-surgical procedures involved in the periocular region are listed. Created in BioRender by Zain Abdin (2025) (https://BioRender.com/rbvun3n)

The periocular region often shows early signs of aging and can benefit significantly from a combination of surgical and non-surgical treatments (Figure 4). These may include fat repositioning or excision, midface or brow lifting, skin resurfacing, and filler use to address periorbital hollows [15].

In terms of surgical technique, endoscopic facelifts have gained popularity due to minimal access incisions and shorter recovery times. It has been demonstrated that a multiplanar dissection technique yields a 93.6% satisfaction rate among patients undergoing frontal and temporal facelifts, with minor complications resolving spontaneously [19].

Minor complications included dimpling at the suture site, asymmetry, overcorrection, transitory paralysis, late edema, hematoma, infection, scarring, and hair loss. However, these complications resolved spontaneously and were negligible after complete recovery. This technique may replace traditional coronal facelifts for middle-aged individuals who request for frontal and temporal facelifts, as traditional coronal facelifts are characterized by considerably extensive trauma and slow recovery [16].

The propeller myocutaneous flap is another valuable approach, particularly for reconstructing the periorbital region. A small patient series using this technique reported no complications after three, six, and 12 months of follow-up, supporting its efficacy and safety [20].

To evaluate patient satisfaction and outcomes, instruments such as FACE-Q and the SCAR scale have proven valuable. FACE-Q has shown strong validity and reliability in measuring changes in patient-reported aesthetic satisfaction [21], while the SCAR scale effectively quantifies improvements in scar appearance and discomfort following refined plastic surgery techniques [6].

Multiplane forehead shortening surgery offers both aesthetic and sensory advantages, reducing forehead length by an average of 2 cm while preserving the frontalis muscle and supraorbital nerve. Scar visibility was minimal in over 76% of patients [9].

Hairline advancement in the infratemporal region has also been used successfully, particularly in East Asian female patients seeking correction of a wide infratemporal area. All patients in a 19-person series reported improvement in facial proportions [17].

Regarding nose reconstruction surgeries, a North American survey by Voizard et al. identified that caudal septoplasty is more commonly performed by surgeons with an FPRS training background [18]. Out of 413 respondents, 87 were from the Association of Otolaryngology-Head and Neck Surgery of Quebec (ORLQC), 108 were from the Canadian Society of Otolaryngology-Head and Neck Surgery (CSOHNS), and 218 were from the American Association of Facial Plastic and Reconstructive Surgery (AAFPRS). Caudal septoplasty was performed in the vast majority of respondents (n = 3,277; 91.3%), and half (51.3%) of the respondents completed a facial plastic and reconstructive surgery (FPRS) fellowship. A literature review was performed for studies published between 1994 and 2017. The initial database search yielded a selection of 235 records, of which 26 were included in the qualitative synthesis after application of exclusion criteria. The number of patients included varied from two to 703, with a total of 1,880 cases. Only four (15%) articles were included a control group. Studies were classified by the surgical technique described. The most common were the swinging door technique (69.5%), extracorporeal septoplasty (46.7%), cartilage scoring (45.3%), and splinting with bone (25.4%). Despite a vast, diverse surgical techniques, surgeons prefer patient-reported outcome measures (PROM) rather than standardized outcome assessment tools [18].

In facial plastic surgery, the potential applications of AI are vast: from research to workflow and patient evaluation, creating before and after pictures, creating 3D prediction models, and assessing postoperative outcomes. With the advent of generative AI (including generative adversarial networks and diffusion models) such as DALL-E, one can create various types of synthetic images using AI. This is very useful for simulating post-surgery result images virtually even before the procedure. Its use in surgical training, in particular, AI-based simulation, is another application that is gaining attention, as algorithmic analyses of video-recorded surgeries can help trainees identify technical weaknesses and predict outcomes of different surgeries. With the use of wearable sensors, an ML algorithm was able to identify movement patterns associated with the skill level of a surgeon. While research using AI is still growing, it could be a tool to produce new ideas for systematic reviews involving a variety of topics in plastic surgery [19].

Lin and Yarholar showcased 3D printing’s role in craniomaxillofacial reconstruction. Patient-specific models of orbital fractures improved surgical precision, reducing revision rates by 22%. In one case, a 3D-printed titanium mandible implant restored both form and function in a patient with osteoradionecrosis. Emerging bioprinting technologies now aim to deposit living cells into scaffolds, enabling in situ regeneration of skin and cartilage [20].

The oncological safety of bioengineered constructs is another critical area for future investigation. Current limitations include the lack of dermal appendages, incomplete tissue functionality, and risks related to growth factors and scaffold materials [8]. Long-term integration and stability of tissue-engineered constructs must also be addressed through targeted research [5].

However, it should be noted that one difficulty with developing models is the significant variation in facial anatomy between individuals, which necessitates a large number of sample images to train AI-based algorithms. Another major concerns are ethical and legal implications; the use of AI under the same ethical principles dictated by traditional human-provided care is not fully addressed by the fact that the increasing involvement of advanced technology in healthcare presents unique questions that may not be fully answered by traditional ethical principles [19].

The COVID-19 pandemic brought additional challenges, limiting surgical access to life- or vision-threatening conditions and highlighting the need for adaptable, resilient systems of care [21]. Padley and Di Pace linked pandemic-induced remote work to a 45% surge in cosmetic consultations, driven by prolonged self-viewing on video calls. Procedures such as blepharoplasty and chin augmentation saw the highest demand, reflecting desires to enhance “screen-ready” features [22].

Despite these technical advances, global disparities persist. A review by Stanford-Moore et al. found that only 31.1% of LMIC-focused FPRS research included first authors from LMICs, and only 25.2% had senior authors from such regions. Structured partnerships prioritizing LMIC leadership are essential for ensuring equitable development and implementation of reconstructive innovations [4].

It highlights its potential for creating patient-specific engineered tissues, reducing immune rejection, and enhancing reconstructive outcomes. A further limitation that should be analyzed in future studies might be the oncological safety of the components used for tissue engineering, such as GFs, cell types, and scaffolds. Due to the absence of dermal appendages such as sebaceous glands, hair follicles, and neurovascular structures, skin substitutes are yet unable to recreate fully functional skin layers. Substantial progress is still needed in all areas of TE to translate functional TE applications from bench-to-bench side [8].

Infratemporal hair grafting involves transplantation of hair follicles and making an incision accordingly for patients, which addresses concerns regarding close eye sets. It proportionates the facial features accordingly [23].

T-genioplasty and single-piece segment lateralization need resection of the chin segment and its lateral repositioning. It is useful for patients with chin asymmetry and male-to-female transition procedures. There is significant feminization as an outcome [24].

An expectation of perfect and close to ideal outcomes is attributed not only to aesthetic but also to reconstructive surgery. Striving for perfection and excellence in plastic surgery should be regarded as obvious and fully understandable. Good results of treatment are highly expected and, even in extensive reconstructive procedures, are taken almost for granted. In the field of plastic and reconstructive surgery, there are many new techniques, which can recreate artificial tissues or organs with better functionality and structure, providing a promising solution for the remodeling of organs and tissues; they are summarized in Table 1 [25-27].

**Table 1:** Summary of the procedures, indications, and significance of the methods used in facial reconstructive surgery HiPSCs: human-induced pluripotent stem cells, RF: radiofrequency

Technique/method	Procedure	Indications	Significance
Tissue engineering/regenerative medicine	It uses natural or artificial scaffolding, growth factors, gene manipulation, and stem cells to reconstruct several tissues.	It can be used instead of the traditional grafting methods for craniofacial reconstruction.	This technique gives access to newer inventive options to restore function and form [8].
Decellularization-recellularization	Removal of cellular antigens and obtaining of decellularized extracellular scaffolding.	It can be used in procedures requiring preservation of vascular networking.	Aids in promoting regeneration of diseased tissues and improves functionality [5].
Acellular dermal matrix	Involves a dermal graft that uses the epidermis, and other cellular components are eliminated to prevent tissue rejection.	It can be applied to minor procedures/surgeries.	The major benefits are that it reduces tissue rejection and improves recovery of wounds [16].
Propeller myocutaneous flap	Uses the orbicularis oculi muscle along with the overlying skin and preserves the vascular supply.	It is an alternative to standard upper eyelid reconstructive techniques.	It involves no complications [20].
Blepharoplasty	Involves multiple procedures such as repositioning or transfer, mid facelift, skin resurfacing, and fat excision.	Useful for minimally invasive procedures.	Significantly decreases early signs of aging of the upper and mid face [15].
Endoscopic facelift	Performed through smaller incisions.	Useful in procedures requiring quick recovery and minimally invasive surgeries.	Compared to traditional facelifts, it is less traumatic with a faster recovery [19].
Multiplane forehead shortening	Uses multiple planes for reducing the forehead length, as well as preserving the frontalis muscle.	Implied for patients with a long forehead.	This method gives an aesthetic advantage by minimizing the visibility of the scar in most patients [9].
3D face reconstruction	It uses 3D models, guides, splints, and implants that are patient-specific.	This can be applied to modify the structures of the skull.	Comparatively needs lesser operative time and gives better aesthetic outcomes [18].
HiPSCs	These cells are similar to embryonic stem cells and can be isolated to regenerate tissue.	It assists in the regeneration of soft tissue and bone.	Enhances the clinical application of HiPSCs [7].
Subsurface radiofrequency	Use of RF treatments in plastic surgery.	It can be an alternative to traditional surgery.	Helpful in the research of RF treatments [11].

## Conclusions

Facial reconstruction surgery is no longer confined to the realm of aesthetics and proportion; it is the very essence of the expansion frontiers of modern medicine. This paper highlights the role of bioprinting, regenerative medicine, and artificial intelligence (AI) in making procedures that were once deemed impossible possible. Nonetheless, the advancement in technology is not the only factor that is needed. Innovation is just a pile of unused data if it is costly, unregulated, or untested in real clinical practice.

The challenge of choice is not the absence of options; these are easily obtainable. The real test lies in making them safe, scalable, and available for all patients who require them. This paper stresses the importance of cooperation between surgeons, researchers, and policymakers to put into practice groundbreaking science and, in turn, provide useful and important results that can improve patients’ lives. The future of reconstructive surgery relies on the integration of research into clinical practice. Hence, the success of facial reconstruction cannot be ascribed to the sophistication of the technology employed but to how many lives it enhances. This responsibility is clear: we must not only try to determine the limits of the possible but also make sure that the advances we make are the standard of care worldwide.

## References

[1] Singer R, Papadopoulos T (2024). There is no universal standard of beauty. Aesthetic Plast Surg.

[2] Cook R, Eggleston A, Over H (2022). The cultural learning account of first impressions. Trends Cogn Sci.

[3] Devgan L, Singh P, Durairaj K (2019). Surgical cosmetic procedures of the face. Otolaryngol Clin North Am.

[4] Stanford-Moore GB, Canick J, Kaplan S, Lee WT (2023). International collaboration trends in facial plastic and reconstructive surgery: a systematic bibliometric scoping review. JAMA Otolaryngol Head Neck Surg.

[5] Shang Y, Wang G, Zhen Y (2023). Application of decellularization-recellularization technique in plastic and reconstructive surgery. Chin Med J (Engl).

[6] Wu Y, Hua Z, Xiang Y, Zhu S, Chen W, Wei P (2023). Evaluation of facial trauma scars after treating by refining plastic surgery techniques: a follow-up study. J Craniofac Surg.

[7] Hadzimustafic N, D'Elia A, Shamoun V, Haykal S (2024). Human-induced pluripotent stem cells in plastic and reconstructive surgery. Int J Mol Sci.

[8] Borrelli MR, Hu MS, Longaker MT, Lorenz HP (2020). Tissue engineering and regenerative medicine in craniofacial reconstruction and facial aesthetics. J Craniofac Surg.

[9] Ahn YS, Park YY, Chang JW (2019). Multiplane forehead shortening: sparing the frontalis muscle and supraorbital nerve. Plast Reconstr Surg.

[10] Michelle L, Torabi SJ, Hutchison DM, Beumer W, Wong BJ (2023). Standardizing a compact medical photography system for use in facial plastic surgery. Facial Plast Surg Aesthet Med.

[11] Swanson E (2022). A systematic review of subsurface radiofrequency treatments in plastic surgery. Ann Plast Surg.

[12] Kobus K, Kobus-Zaleśna K (2018). Remarks on perfection in plastic surgery of the face. Biomed Res Int.

[13] Marinozzi S, Sanese G, Messineo D, Raposio E, Codolini L, Carbonaro R, Cervelli V (2021). The art of rhinoplasty: researching technical and cultural foundations of Western world rhinosurgery, from the Middle Ages to the Renaissance. Aesthetic Plast Surg.

[14] Oliveira-Santos T, Baumberger C, Constantinescu M, Olariu R, Nolte LP, Alaraibi S, Reyes M (2013). 3D face reconstruction from 2D pictures: first results of a web-based computer aided system for aesthetic procedures. Ann Biomed Eng.

[15] Naik M (2013). Blepharoplasty and periorbital surgical rejuvenation. Indian J Dermatol Venereol Leprol.

[16] Hu X, Ma H, Xue Z, Qi H, Chen B (2017). Endoscopic facelift of the frontal and temporal areas in multiple planes. Singapore Med J.

[17] Park JH (2016). Masking the close eye appearance in the East Asian female population: infratemporal hairline reduction with hair grafting. Aesthetic Plast Surg.

[18] Voizard B, Theriault M, Lazizi S, Moubayed SP (2020). North American survey and systematic review on caudal Septoplasty. J Otolaryngol Head Neck Surg.

[19] Choi E, Leonard KW, Jassal JS, Levin AM, Ramachandra V, Jones LR (2023). Artificial intelligence in facial plastic surgery: a review of current applications, future applications, and ethical considerations. Facial Plast Surg.

[20] Lin AY, Yarholar LM (2020). Plastic surgery innovation with 3D printing for craniomaxillofacial operations. Mo Med.

[21] Langer PD, Bernardini FP (2020). Oculofacial plastic surgery and the COVID-19 pandemic: current reactions and implications for the future. Ophthalmology.

[22] Padley RH, Di Pace B (2021). Touch-ups, rejuvenation, re-dos and revisions: remote communication and cosmetic surgery on the rise. Aesthetic Plast Surg.

[23] Mihalečko J, Boháč M, Danišovič Ľ, Koller J, Varga I, Kuniaková M (2022). Acellular dermal matrix in plastic and reconstructive surgery. Physiol Res.

[24] Hernandez-Boussard T, Bozkurt S, Ioannidis JP, Shah NH (2020). MINIMAR (MINimum Information for Medical AI Reporting): developing reporting standards for artificial intelligence in health care. J Am Med Inform Assoc.

[25] Ouyang Y, Wang K, Zhang T, Peng L, Song G, Luo J (2020). The influence of sports participation on body image, self-efficacy, and self-esteem in college students. Front Psychol.

[26] Delia G, Fazio A, Parafioriti A, Meduri A, Inferrera L, Stagno d'Alcontres F (2021). The propeller myocutaneous flap of the upper eyelid: anatomical study and its clinical implication. J Craniofac Surg.

[27] Pokrowiecki R, Šufliarsky B, Jagielak M (2024). Esthetic surgery of the chin in cis- and transgender patients-application of T-genioplasty vs. single-piece segment lateralization. Medicina (Kaunas).

